# Hemoglobin-associated *CALR* in proximal tubule cells can be used as a biomarker for idiopathic membranous nephropathy

**DOI:** 10.3389/fmed.2025.1574852

**Published:** 2025-06-11

**Authors:** Shuting Pang, Qiuyan Tan, Boji Xie, Bingmei Feng, Rongbin Zhou, Zige Liu, Haiyuan Wei, Yian Huang, Mujia Jili, Yuli Xie, Shanshan Li, Binran Zhao, Wei Li, Rirong Yang

**Affiliations:** ^1^Department of Nephrology, The Second Affiliated Hospital of Guangxi Medical University, Nanning, China; ^2^Center for Genomic and Personalized Medicine, Guangxi Key Laboratory for Genomic and Personalized Medicine, Guangxi Collaborative Innovation Center for Genomic and Personalized Medicine, University Engineering Research Center of Digital Medicine and Healthcare, Guangxi Medical University, Nanning, China; ^3^Institute of Urology and Nephrology, The First Affiliated Hospital of Guangxi Medical University, Guangxi Medical University, Nanning, China; ^4^Department of Immunology, School of Basic Medical Sciences, Guangxi Medical University, Nanning, China

**Keywords:** idiopathic membranous nephropathy, proximal tubular cells, *CALR*, urine cell RNA sequencing, urine biomarkers

## Abstract

**Objective:**

Idiopathic membranous nephropathy (IMN) is the leading cause of nephrotic syndrome in adults. Given the limited diagnostic options currently available, we performed RNA sequencing (RNA-seq) of urinary cells to identify potential urinary biomarkers for IMN and to investigate its underlying disease mechanisms.

**Methods:**

We conducted RNA-seq analysis on cells isolated from both first-void and second-void morning urine samples. By integrating these data with single-cell RNA sequencing (scRNA-seq) data from IMN and healthy kidney tissues, we performed comprehensive analyses including: Inflammatory index assessment, Kyoto Encyclopedia of Genes and Genomes (KEGG) pathway analysis, Gene Ontology (GO) functional annotation, Gene Set Enrichment Analysis (GSEA) enrichment analysis, Protein-Protein Interaction (PPI) network construction. Candidate differentially expressed genes (DEGs) were further validated using urinary cell RNA-seq data. Key genes were ultimately identified through Weighted Gene Co-expression Network Analysis (WGCNA) and subsequently verified by immunohistochemical and Quantitative Real-Time PCR (qRT-PCR) experiments of tissue expression patterns.

**Results:**

Our findings demonstrate that the *CALR* gene is significantly associated with IMN pathogenesis and progression. Functionally, *CALR* plays crucial roles in both immune response (particularly in antigen presentation) and physiological processes (notably in hemoglobin production).

## 1 Introduction

Idiopathic membranous nephropathy (IMN), also known as primary membranous nephropathy, accounts for approximately 30% of adult nephrotic syndrome cases (1), with about 40% of IMN patients progressing to end-stage renal disease (2, 3). Early detection and diagnosis of IMN enables timely treatment initiation and reduces the economic burden on both families and society. For a considerable period, renal biopsy has been the gold standard for IMN diagnosis (4). However, this invasive procedure carries multiple contraindications and potential complications, including bleeding risk (5). Moreover, renal biopsy only provides a static snapshot of the disease status and cannot facilitate dynamic monitoring of disease progression or prognosis (3, 4, 6). The pathogenesis of IMN is closely associated with Human Leukocyte Antigen molecules, complement system activation, and autoantigen-antibody interactions (7–9). Recent mechanistic studies have identified several diagnostic biomarkers for IMN, including anti-PLA2R and anti-THSD7A antibodies, which directly target disease-specific autoantigens involved in the immunopathogenesis of this condition (10, 11). Subsequent studies demonstrated that the anti-PLA2R antibody test achieved a sensitivity of 0.64 and specificity of 0.99 for IMN diagnosis, showing comparable diagnostic accuracy to renal biopsy (12). These findings suggest that appropriate biomarkers may complement renal biopsy in IMN diagnosis and evaluation.

Urine collection is a simple and non-invasive procedure. Since urinary components are primarily derived from kidney metabolism, they are less affected by other systemic activities (13). Notably, urine contains various exfoliated renal cells, including proximal tubular cells (PTCs) and podocytes, which hold significant research value (14). Studies have demonstrated that adequate quantities of PTCs are shed in urine from both healthy individuals and patients with kidney disease, making them reliable indicators of renal function changes (15). Different urine sampling methods offer distinct advantages. First-morning urine, the most commonly used clinical sample, is convenient to collect. Importantly, the second morning urine specimen (collected after the initial void) shows an increased proportion of renal cells. This sampling approach helps minimize contamination by epithelial cells and other interfering factors, thereby providing a more accurate representation of kidney pathology. RNA sequencing technologies offer powerful analytical capabilities. While bulk RNA-Seq (RNA-seq) provides comprehensive transcriptome profiling, single-cell RNA sequencing (scRNA-seq) enables large-scale, unbiased characterization of individual cells at genome-wide scale (16). scRNA-seq analysis of urine samples from diabetic nephropathy patients revealed a remarkably high correlation (approaching 1:1 correspondence) between urinary PTCs and their renal tissue counterparts. These findings demonstrate that urinary PTCs can faithfully mirror functional alterations in renal tissue PTCs, establishing them as valuable sources for non-invasive kidney disease diagnostics (17).

Proximal tubular cells (PTCs) play a pivotal role in renal reabsorption, secretion, excretion, and water-electrolyte homeostasis (18). Recent in-depth studies have revealed that PTC injury not only accompanies but actively drives disease progression, ultimately contributing to renal dysfunction (19). The analysis of urinary PTCs shows great potential for identifying novel biomarkers of IMN. By integrating scRNA-seq of IMN renal tissues with urine cell RNA-seq, we identified calreticulin (*CALR*) in PTCs as a critical regulator of IMN pathogenesis.

Calcium ion homeostasis plays a pivotal regulatory role in the growth and development of renal cells. Studies have demonstrated that Klotho protein, which is closely associated with calcium ion regulation, serves a crucial function in maintaining podocyte cytoskeletal stability in IMN (20). *CALR* is a calcium-binding protein with a molecular weight of 46 kDa. It plays crucial roles in multiple biological processes, including regulation of cellular calcium homeostasis, protein folding facilitation, modulation of cell motility, metabolic regulation, gene expression control, cell cycle regulation, and apoptosis (21–23). During embryonic development, complete *CALR* knockout results in cardiac and renal developmental failure, ultimately leading to embryonic lethality, demonstrating its essential role in normal organismal development (24, 25). In addition, aberrant expression and dysfunction of *CALR* can disrupt calcium homeostasis through multiple mechanisms, including: (1) dysregulation of oxidative stress pathways (e.g., AMPK signaling); (2) activation of mitophagy and NF-κB signaling pathway; and (3) significant correlations with renal inflammatory responses and tissue fibrosis progression (26, 27).

In this study, we mined genes expressed in various cell populations of patients with IMN through renal tissue scRNA-seq combined with urinary cellular RNA-seq in morning and second-void morning urine, with a focus on PTCs. We found that *CALR* is closely associated with IMN progression and can be used as a biomarker for IMN.

## 2 Research methods and materials

### 2.1 Collection of samples and clinical information

This study enrolled 17 PLA2R-positive IMN patients and 17 healthy volunteers as study participants. Renal biopsy results and PLA2R antibody status served as diagnostic criteria for membranous nephropathy classification.

Exclusion criteria included:(1) anuric or dialysis-dependent patients; (2) patients with severe systemic diseases or major organ dysfunction; (3) patients who underwent renal biopsy within the preceding 3 months; (4) patients with poor medical compliance. The control group comprised 17 healthy volunteers. The study protocol was approved by the Institutional Review Board of the Second Affiliated Hospital of Guangxi Medical University (Approval No. 2023KY-0715) and conducted in compliance with the ethical principles of the Declaration of Helsinki. Written informed consent was obtained from all participants or their legal guardians.

The first-void morning urine (i.e., morning urine), was collected on the day following the patient’s admission to the hospital. Subsequently, a second-void morning urine, that is secondary morning urine, was collected within 2 h. After collection, the urine was immediately stored at 4°C and centrifuged at 490 × g for 10 min (Eppendorf, Hamburg, Germany). The supernatant was aliquoted into 1.5 mL EP tubes (Axygen, California, the United States). The cell pellet was washed by resuspension in pre-cooled DPBS (Wisent, Montreal, Canada) and centrifuged at 2,000 rpm for 5 min at 4°C (Eppendorf, Hamburg, Germany). After discarding the supernatant, the washing step was repeated to obtain the final urine cell pellet, which was stored at −80°C (Thermo Fisher Scientific, Massachusetts, the United States).

Clinical information of patients with IMN and healthy volunteers, such as hemoglobin level, urinary creatinine, renal function, uric acid, and blood lipids, was collected. For healthy individuals, clinical information was obtained from the results of the physical examinations at the Second Affiliated Hospital of Guangxi Medical University. Patients, the information was retrieved from their medical records.

### 2.2 Urine cell RNA-seq and primary analysis of raw sequencing data

The cDNA library was constructed from urine cell RNA (stored at −80°C) using the Singleron AccuraCode^®^ HTP OneStep RNAseq Kit (Singleron, Nanjing, China) following the manufacturer’s protocol. After library preparation, stringent quality control (QC) was performed, including assessments of total library yield (> 30 ng), fragment size distribution (main peak: 300–600 bp), large fragment contamination (900–5,000 bp fragments < 20%), and small fragment residues (< 300 bp fragments < 20%). Qualified libraries were subjected to paired-end sequencing on the Illumina NovaSeq platform.

Raw reads were processed using Celescope (v2.0.7) with default parameters to generate gene expression profiles. Briefly, barcodes and unique molecular identifiers (UMIs) were extracted from R1 reads and error-corrected. Adapter sequences and poly-A tails were trimmed from R2 reads using cutadapt (v3.7). The trimmed R2 reads were then aligned to the Homo sapiens GRCh38.99 reference genome with STAR (v2.7.11a). Uniquely mapped reads were assigned to genes using featureCounts (v2.0.1). Finally, successfully assigned reads sharing identical barcodes, UMIs, and gene annotations were aggregated to generate the gene expression matrix for downstream analysis.

### 2.3 Single-cell data sources and cell clustering

scRNA-seq data from nine patients with IMN and six healthy adults were obtained from the Gene Expression Omnibus (GEO) database (accessions: GSE241302, GSE171458, and GSE131685).

All analyses were performed using R software (version 4.3.1).^1^ Dataset integration was conducted with the Harmony package. QC was applied via the Seurat package to exclude cells expressing fewer than 500 genes, more than 7,000 genes, or exceeding 20% mitochondrial read content. After QC, 44,363 cells were retained for downstream analysis. Cell clustering was performed using the “FindClusters” function (resolution = 0.8), with the top five marker genes defining cell identity. Clusters were visualized using t-distributed stochastic neighbor embedding (t-SNE).

### 2.4 Inflammation score evaluation

The inflammation score was evaluated by quantifying the expression of inflammation-related genes. These genes—including *IFNG*, *IFNGR1*, *IFNGR2*, *IL10*, *IL12A*, *IL12B*, *IL12RB1*, *IL12RB2*, *IL13*, *IL17A*, *IL17F*, *IL18*, *IL18R1*, *IL18RAP*, *IL1A*, *IL1B*, *IL2*, *IL21*, *IL21R*, *IL22*, *IL23A*, *IL23R*, *IL2RG*, *IL4*, *IL4R*, *IL5*, *IL6*, *JUN*, *NFKB1*, *RELA*, *RORA*, *RORC*, *S100A8*, *S100A9*, *STAT1*, *STAT3*, *STAT4*, *STAT6*, *TGFB1*, *TGFB2*, *TGFB3*, and *TNF*—were obtained from a previous study (28).

### 2.5 Identification and functional enrichment of DEGs

We performed batch effect correction on urine urothelial cells RNA-seq data using the SVA package. Differentially expressed genes (DEGs) were identified through stringent dual criteria: absolute log2 fold-change (|log2FC|) ≥ 1 (corresponding to ≥ 2-fold expression difference) with Benjamini-Hochberg adjusted *p*-value (adj.P.Val) < 0.05 (controlling false discovery rate [FDR] ≤ 5%). Differential expression analysis was conducted using the limma R package, which employs linear models suitable for both microarray and RNA-seq data. The analytical workflow consisted of constructing a design matrix (model.matrix) reflecting experimental groupings, fitting linear models to expression data using lmFit, defining treatment-control contrasts through makeContrasts and applying them with contrasts.fit, and moderating standard errors of log2FC estimates using empirical Bayes methods (eBayes) to enhance accuracy for low-expression genes. This analysis identified 5,662 DEGs in morning urine urothelial cells RNA-seq and 5,936 DEGs in second morning urine urothelial cells RNA-seq samples.

Subsequent functional enrichment analysis of DEGs was performed using clusterProfiler, focusing on Gene Ontology (GO) terms and Kyoto Encyclopedia of Genes and Genomes (KEGG) pathway annotations. Gene Set Enrichment Analysis (GSEA) was also implemented through the same package, with all genes ranked in descending order based on their log2 fold-change values. Pathways with *p* < 0.05 were considered statistically significant and retained for further analysis.

### 2.6 Construction of PPI network and screening of DEGs

DEGs in PTCs were initially screened. Gene importance scores were calculated and ranked in Cytoscape (v3.8.2) using three topological analysis methods: maximum clique centrality (MCC), molecular complex detection (MCODE), and Degree. Each gene was independently evaluated by all three methods. The top 50 candidate genes from the DEGs were selected, and their overlaps were visualized via a Venn diagram generated with the R package VennDiagram. Ultimately, eight core DEGs were identified as key regulators.

### 2.7 Verification of the expression of DEGs through urinary cellular RNA-seq

The heatmap of DEGs was generated using the heatmap function in R. Violin plots, boxplots, and scatterplots were subsequently created with the ggplot2 package. The diagnostic potential of these DEGs was evaluated by receiver operating characteristic (ROC) curve analysis, where a larger area under the curve (AUC) value indicates higher diagnostic sensitivity for the corresponding gene.

### 2.8 WGCNA on urine cells in secondary morning urine

The Weighted Gene Co-expression Network Analysis (WGCNA) package was utilized to investigate associations between gene co-expression patterns and phenotypic variations. Highly correlated genes were clustered into modules, and module-trait relationships were analyzed to identify hub genes potentially driving phenotypic changes. An unsigned network topology was adopted for construction. Gene-gene correlations were assessed using Pearson’s correlation coefficients. Finally, the overlap between these hub genes and the previously identified DEGs was visualized via Venn diagram analysis to pinpoint the most robust diagnostic candidates.

### 2.9 Immunohistochemistry

Renal tissue sections were obtained from the Second Affiliated Hospital of Guangxi Medical University with informed consent from donors or their families, and the study protocol was approved by the Institutional Ethics Committee (Approval No. 2023KY-0715). Immunohistochemistry was performed using a commercial kit (Proteintech, Chicago, the United States) according to the manufacturer’s protocol. Briefly, after deparaffinization and heat-induced antigen retrieval with EDTA (Solarbio, Beijing, China), endogenous peroxidase activity was quenched with 3% H_2_O_2_ (10 min, room temperature). Sections were blocked with kit-provided buffer, then incubated overnight at 4°C with rabbit anti-CLAR primary antibody (1:50 dilution; Abways, Shanghai, China) in a humidified dark chamber. Following TBST washes (3 × 5 min; Solarbio, Beijing, China), HRP-conjugated secondary antibody was applied (30 min, room temperature). Signal detection used DAB chromogen with hematoxylin counterstaining, followed by ethanol dehydration and xylene-based mounting with neutral resin (Solarbio, Beijing, China). Quantitative analysis was performed using ImageJ (v1.53; NIH, the United States).

### 2.10 Quantitative real-time PCR (qRT-PCR) analysis in renal tissues

Renal tissues from patients with IMN and histologically confirmed paired adjacent normal tissues were procured from the Second Affiliated Hospital of Guangxi Medical University. Sample collection complied with ethical regulations, including informed consent from donors/legal representatives and approval by the Institutional Ethics Committee (Approval No.2025KY-0099).

Total RNA was isolated using FastPure^®^ Cell/Tissue Total RNA Isolation Kit V2 (Vazyme, Nanjing, China). cDNA synthesis employed PrimeScript™ RT Master Mix (TaKaRa, Kyoto, Japan). qRT-PCR was subsequently performed using FastStart Essential DNA Green Master (Roche, Basel, Switzerland) following the manufacturer’s protocol. β*-Actin* served as the endogenous reference gene.

Relative gene expression levels were calculated using the 2^–ΔΔCt^ method and analyzed with GraphPad Prism 9.0 (GraphPad Software, United States). The quantitative experimental data are presented as mean ± standard deviation (Mean ± SD). Independent samples *t*-tests were used for comparisons between two groups, while one-way analysis of variance (One-way ANOVA) was employed for multi-group comparisons. Statistical significance was defined as *P < 0.05, **P < 0.01, ***P < 0.001, and ****P < 0.0001. The primer sequences for β*-actin* and *CALR* are listed in Supplementary Table 1 (GenSys biotechnology, Nanning, China).

## 3 Results

### 3.1 Single-cell atlas of IMN and healthy kidney tissues

**FIGURE 1:**
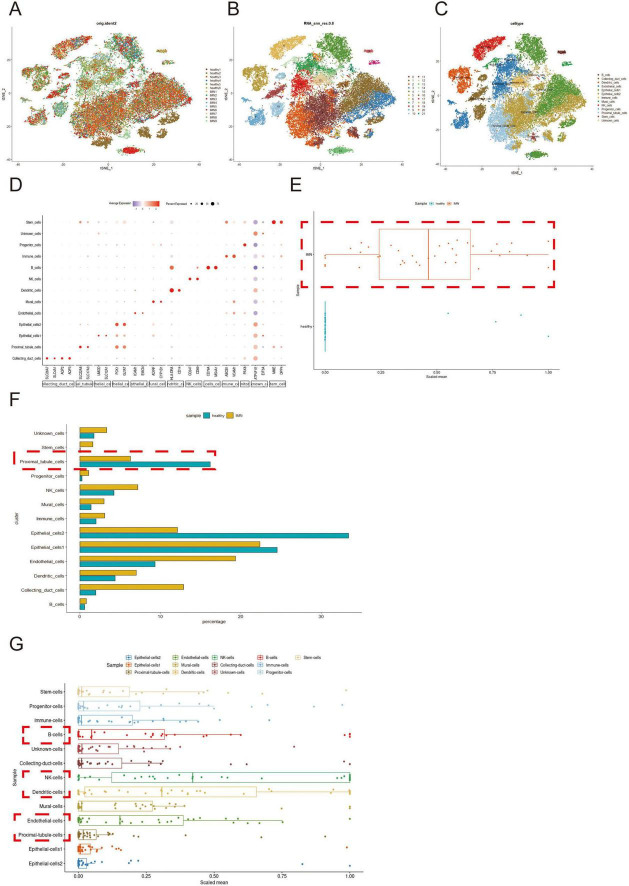
Single-cell profiling and inflammatory signature analysis of renal tissues in IMN versus normal controls. **(A,B)** scRNA-seq datasets from IMN and normal groups were integrated using the Harmony package (color-coded by sample origin). Unsupervised clustering based on highly variable genes identified 21 initial cell clusters. **(C,D)** After batch correction, 13 distinct cell types were resolved, with cluster-specific marker genes visualized by bubble plots (dot size: detection rate; color intensity: mean expression). **(E)** Comparative analysis of inflammatory gene expression (box plots: center line, median; box limits, Interquartile Range). **(F)** Differential abundance of major cell populations between groups (box plots). **(G)** Cell-type-specific inflammatory scores of patients with IMN were computed from curated gene signatures. Higher-resolution versions of **(A–C)** are provided in Supplementary Figures 2–4.

After quality control and integration of scRNA-seq data from nine IMN patients and six healthy donors, 44,363 high-quality cells were retained for analysis (Supplementary Figure 1). Unsupervised clustering of variably expressed genes via principal component analysis revealed 21 initial clusters, which were consolidated into 13 major cell types after batch correction. These comprised: Collecting duct cells, endothelial cells (subtypes 1-2), epithelial cells (subtypes 1-2), mural cells, and proximal tubular cells (PTCs), B cells, dendritic cells, NK cells, progenitor cells, stem cells, and unclassified immune cells (Figures 1A–C).

Cluster-specific marker genes were visualized through bubble plots, where dot size reflected gene detection rate and color intensity indicated normalized expression levels (Figure 1D). Comparative analysis demonstrated significantly elevated inflammatory gene expression in IMN versus healthy tissues (Figure 1E), corroborated by increased proportions of immune and stromal cell populations (mural cells, endothelial cells 2, etc.) in IMN (Figure 1F).

Notably, although PTCs were depleted in IMN tissues, they exhibited marked inflammatory activation (Figure 1G), suggesting their potential role in initiating immune responses. Cell-type-specific inflammatory scores implicated B cells, NK cells, dendritic cells, endothelial cells, and PTCs as key contributors to IMN-associated inflammation.

### 3.2 Functional enrichment analysis of DEGs in PTCs

**FIGURE 2:**
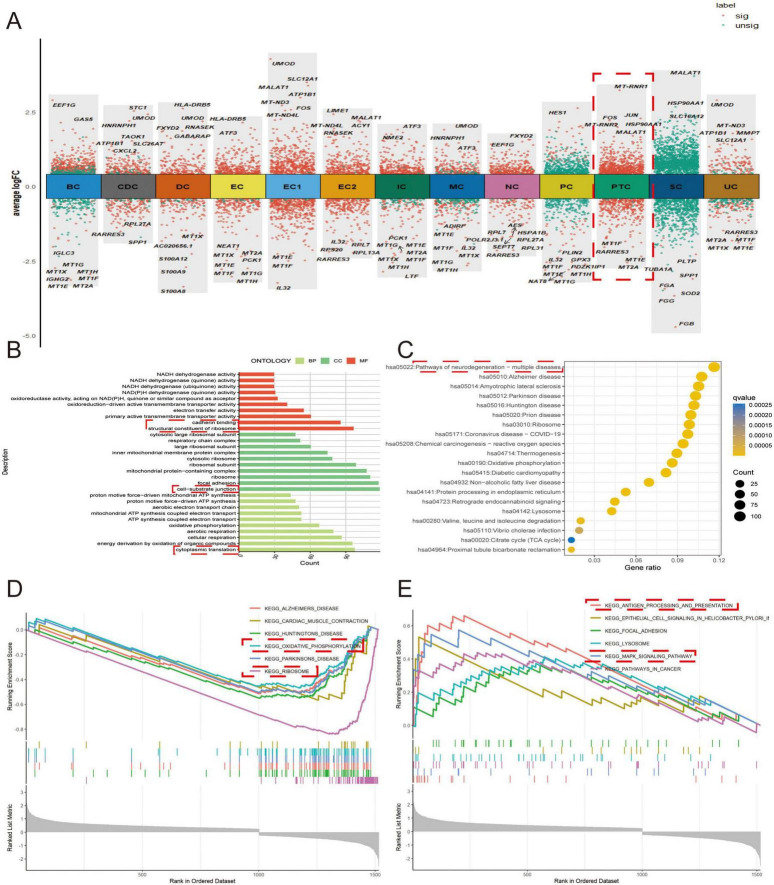
Functional enrichment analysis of DEGs in PTCs. **(A)** Volcano plots displaying DEGs across all cell types. **(B)** DEGs from proximal tubular epithelial cells were selected for GO analysis. BP refers to biological process, CC indicates cellular composition, and MF stands for molecular function. **(C)** Disease-associated pathways identified by KEGG analysis. **(D,E)** GSEA of **(D)** downregulated and **(E)** upregulated gene sets.

After identifying the DEGs of each cell cluster, we selected the DEGs of PTCs for functional enrichment to explore the role of PTCs in IMN (Figure 2A). GO analysis suggested that the DEGs were associated with cellular substrate junctions, ribosomal structures, calcineurin binding, and cytoplasmic translation. KEGG analysis suggested that the DEGs were associated with various diseases (Figures 2B,C). For further analysis, we subjected the up-regulated and down-regulated DEGs to functional enrichment analyses. Through GSEA, we found that the down regulated DEGs were associated with oxidative phosphorylation and ribosome function. The up-regulated DEGs were associated with antigen processing and presentation, and the mitogen-activated protein kinase (MAPK) signaling pathway (Figures 2D,E). This suggests that PTCs play a major role in the activation of inflammatory signaling pathways, which is consistent with the finding of Jiang et al. (29).

### 3.3 Protein-protein interaction network construction to identify key pathogenic genes

**FIGURE 3:**
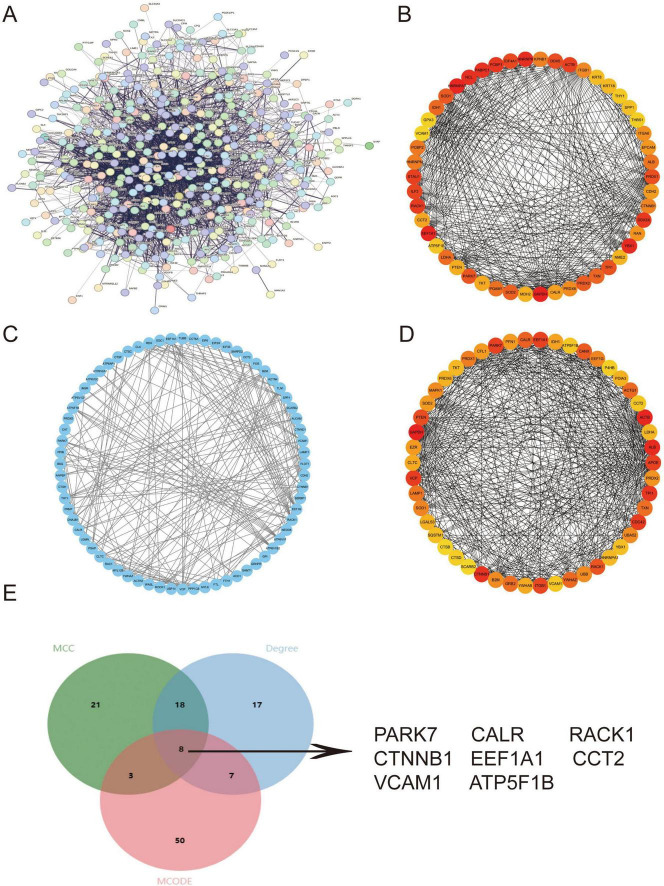
PPI network construction to identify key pathogenic genes. **(A)** PPI network constructed from the intersection of PTC DEGs and extracellular protein-encoding genes in the HPA database. **(B–D)** Top 50 hub genes identified through Cytoscape-based topological analyses using **(B)** MCC, **(C)** MCODE, and **(D)** Degree. **(E)** Venn diagram demonstrating eight consensus key genes (*PARK7*, *CALR*, *RACK1*, *CTNNB1*, *EEF1A1*, *CCT2*, *VCAM1*, *ATP5F1B*) derived from the intersection of all three methods and extracellular protein-encoding genes in HPA. Higher-resolution versions of **(A–D)** are provided in Supplementary Figures 5–8.

Given the significantly reduced proportion of PTCs in IMN versus healthy renal tissues, we hypothesized pathological shedding of PTCs into urine. Leveraging the Human Protein Atlas (HPA) database (30), we built PPI networks using the intersection of PTC-specific DEGs and genes encoding extracellular proteins (Figure 3A). Through integrated analysis employing three distinct algorithms—MCC, MCODE and Degree - we identified the top 50 hub genes (Figures 3B,D). This multi-method approach yielded eight high-confidence candidate genes (*PARK7*, *CALR*, *RACK1*, *CTNNB1*, *EEF1A1*, *CCT2*, *VCAM1*, and *ATP5F1B*) for subsequent experimental validation (Figure 3E).

### 3.4 Validation of DEGs and diagnostic efficacy through RNA-seq in secondary morning urine urothelial cells

**FIGURE 4:**
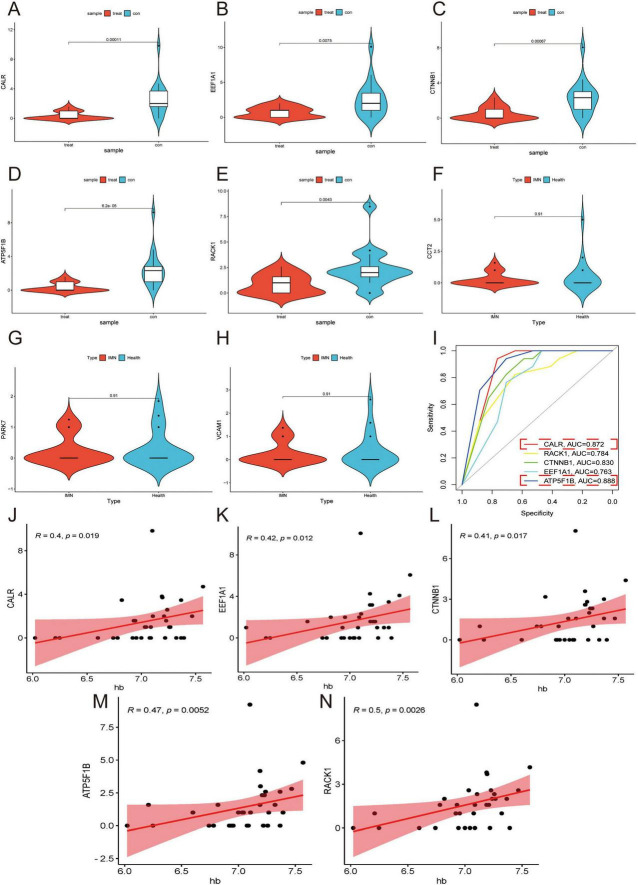
Validation of DEGs in secondary morning urine. **(A–H)** Violin plots are used to demonstrate the validation of the expression of DEGs in secondary morning urine. **(I)** ROC curves are employed to explore the correlation of DEGs. Higher AUC values indicate a higher correlation (AUC > 0.7 considered clinically significant). **(J–N)** Correlation analysis of DEGs with Hb is performed. Higher R values indicate a higher correlation while *p* < 0.05.

Comparative analysis between IMN patients (*N* = 17) and healthy controls (*N* = 17) revealed significant differential expression of *CALR*, *RACK1*, *CTNNB1*, *EEF1A1*, and *ATP5F1B* (*p* < 0.05), while *CCT2*, *PARK7*, and *VCAM1* showed no significant changes (*p* > 0.05) (Figures 4A–H). ROC curve analysis demonstrated strong correlations between IMN and *CALR*, *RACK1*, *CTNNB1*, *EEF1A1*, and *ATP5F1B* expression, with *ATP5F1B* and *CALR* exhibiting the highest diagnostic accuracy (Figure 4I). Correlation studies with clinical parameters (Supplementary Table 2) showed that all five DEGs positively associated with hemoglobin (Hb) levels (*p* < 0.05; Figures 4J–N), while *CALR*, *RACK1*, and *CTNNB1* additionally correlated positively with glomerular filtration rate (GFR). Notably, *RACK1* uniquely showed negative correlation with serum creatinine (Scr), and *CTNNB1* alone inversely correlated with lipid levels (Supplementary Figures 9, 10). These findings strongly suggest that PTC-derived DEGs in urine cells are clinically relevant to Hb regulation and renal function in IMN patients.

### 3.5 Validation of the function of DEGs through RNA sequencing of morning urine urothelial cells

**FIGURE 5:**
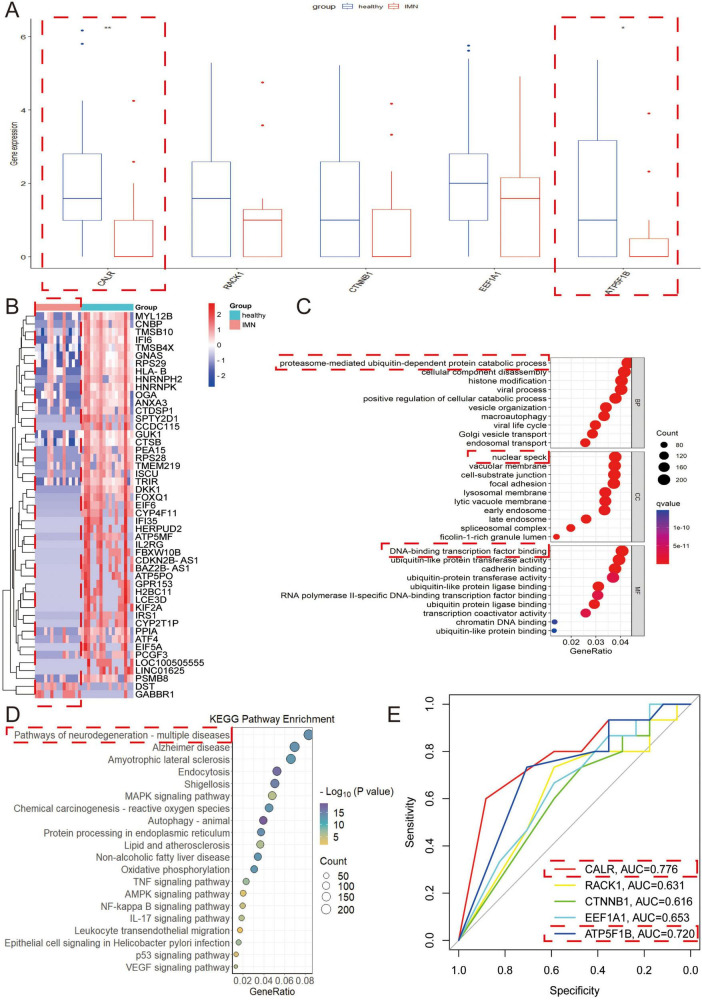
Validation of the function of DEGs through RNA sequencing of morning urine urothelial cells. **(A)** The key genes with differential expression in secondary urine were verified in morning urine. **(B)** Heat map of differential genes in morning urine. Red indicates high expression, and blue indicates low expression. The darker the color, the greater the difference in expression. **(C,D)** The differential genes in morning urine urothelium were selected for GO and KEGG analyses. In the GO analysis, BP refers to biological process, CC denotes cellular composition, and MF represents molecular function. **(E)** ROC curves were used to explore the correlation of each key gene. Higher AUC values indicate greater correlation (AUC > 0.7 considered clinically significant).

We validated the consistency of DEGs through RNA sequencing of morning urine urothelial cells, revealing significant differential expression of *CALR* and *ATP5F1B* in IMN patients (*n* = 15) vs. healthy controls (*n* = 17) (*p* < 0.05), while *RACK1*, *CTNNB1*, and *EEF1A1* showed no significant changes (Figure 5A), suggesting *CALR* and *ATP5F1B* as potential IMN biomarkers due to their consistent dysregulation. Heatmap analysis demonstrated predominant under expression of DEGs in IMN samples (Figure 5B), potentially implicating transcriptional suppression in disease progression, while functional analyses identified associations with proteasome-mediated ubiquitin-dependent protein catabolism, nuclear speckle organization, DNA-binding transcription factor activity, and neurodegenerative disease pathways (Figures 5C,D). ROC curve analysis confirmed diagnostic correlations for all five genes, with *CALR* and *ATP5F1B* exhibiting the highest predictive accuracy (Figure 5E).

### 3.6 Mining for core genes in secondary morning urine cells using Weighted gene co-expression network analysis (WGCNA)

**FIGURE 6:**
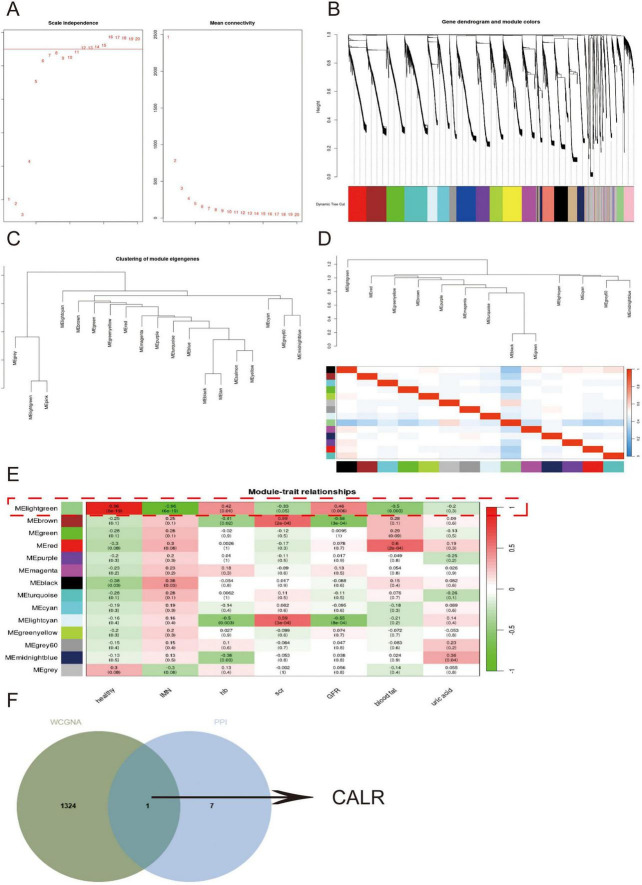
Mining for core genes in secondary morning urine cells using WGCNA. **(A,B)** Demonstration of the selection of soft thresholds and the construction of the network. **(C,D)** Similar modules are merged followed by linkage analysis. **(E)** The correlation of each module with the disease and clinical manifestations. **(F)** Venn diagrams show the intersecting genes of the Melightgreen module genes with the PPI network.

Given that secondary morning urine cells more accurately reflect renal cellular composition than first-void morning urine, we performed WGCNA using RNA-seq data from these cells integrated with clinical information. The gene co-expression network was constructed using an optimal soft threshold, with correlation coefficients calculated from normalized expression profiles. Hierarchical clustering identified 20 initial modules (gray module representing unassigned genes) (Figures 6A,B). After merging similar modules, 14 distinct gene modules were retained for downstream analysis. Module eigengene analysis revealed high interconnectivity among co-expressed modules, with branches in the dendrogram indicating functional similarity (Figures 6C,D). Notably, the MElightgreen module showed significant correlations with IMN diagnosis, hemoglobin levels, and glomerular filtration rate (Figure 6E). Intersection of MElightgreen module genes with PPI network-derived DEGs pinpointed *CALR* as a hub gene (Figure 6F), suggesting its pivotal role in PTC-mediated inflammatory responses during IMN progression.

### 3.7 Verification of *CALR* expression by RNA-seq, immunohistochemistry and qRT-PCR

**FIGURE 7:**
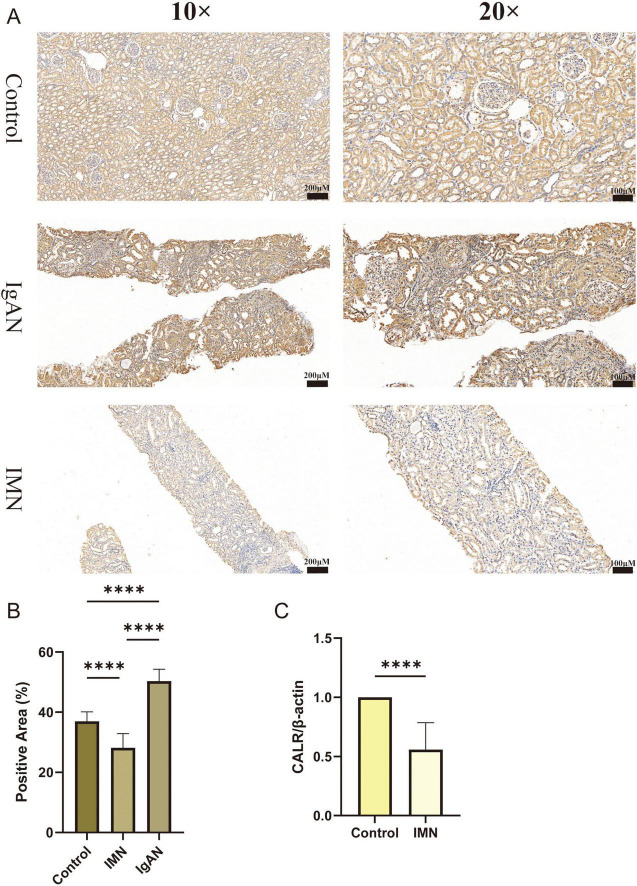
Verification of *CALR* expression by immunohistochemistry and qRT-PCR. **(A,B)** Immunohistochemical results of *CALR* expression in Control, IMN, IgAN groups. **(C)** qRT-PCR results of *CALR* expression in renal tissues from Control and IMN groups. Statistical significance was defined as *P < 0.05, **P < 0.01, ***P < 0.001, and ****P < 0.0001.

To further validate *CALR* expression patterns, we performed immunohistochemistry and qRT-PCR analyses. Immunohistochemistry confirmed reduced *CALR* protein expression in patients with IMN, whereas IgA nephropathy (IgAN) cases showed elevated expression (Figures 7A,B). Independent qRT-PCR validation demonstrated marked downregulation of *CALR* mRNA in IMN compared with normal renal tissues (Figure 7C). This cross-platform consistency establishes reduced *CALR* expression as an IMN-specific signature, supporting it could be a diagnostic biomarker. The immunohistochemical results for secondary MN are presented in Supplementary Figure 11.

## 4 Discussion

IMN is one of the most prevalent forms of glomerulonephritis and the most common cause of proteinuria in clinical practice. Due to its insidious onset, it often leads to severe complications by the time it is detected (31). In the advanced stage, most patients progress to end-stage renal disease and need to rely on renal replacement therapy to sustain their lives, which imposes a serious socio-economic burden (32). The pathogenesis of IMN, an autoimmune disease, remains unclear. However, the emergence of transcriptomics sequencing technology has provided a more comprehensive understanding of IMN (33). To this end, we proposed the use of urine cellular RNA-seq combined with renal tissue scRNA-seq to explore biomarkers for IMN.

The results of scRNA-seq analysis of IMN and normal renal tissues showed that the kidney was mainly divided into 13 major cell populations, including collecting duct cells, PTCs, and various immune cells. Combined with the cell type abundance analysis, it was suggested that the proportion of immune cells in renal tissues should not be overlooked. The expression of inflammatory response genes further corroborates the key role of the immune system in IMN. The inflammatory response gene expression profile scores were evaluated for each of the 13 cell clusters. B cells, endothelial cells, and PTCs exhibited notable inflammatory scores. Studies have demonstrated that PTCs can activate immune cells through secreted factors (34). However, whether PTCs in IMN regulate immune responses and secreted factors they do so remains unknown. Moreover, the role of PTCs in IMN needs to be further explored.

Functional enrichment analysis of the DEGs of PTCs indicated that PTCs were mainly related to cellular substrate attachment, ribosome structure, calcineurin binding, and cytoplasmic translation. Both ribosome structure and cytoplasmic translation are associated with protein formation and the secretory function of PTCs. However, a decrease in *CALR* expression correlated with epithelial cell phenotypic switching. Moreover, the decrease in E-*CALR* expression after stimulation of human renal cortical proximal tubular epithelial cells (HK-2) with TGF-β suggests that *CALR* in PTCs is closely related to renal tubular fibrosis (35, 36). GSEA analysis showed that the down-regulated DEGs were related to oxidative phosphorylation, ribosome function, etc. The up-regulated genes were associated with antigen processing and presentation and the MAPK signaling pathway. Oxidative phosphorylation is a source of cellular energy. In diabetic nephropathy (DN), deficiencies in oxidative phosphorylation reactions lead to energy deficiency in PTCs, which in turn exacerbates DN progression (37). In a study on renal tubulointerstitial fibrosis, podocyte particles were found to promote fibrosis in PTCs by activating of the MAPK inflammatory pathway (38). This indicated that DEGs in PTCs are mainly related to renal tubular fibrosis and play an important role in the generation of inflammatory responses.

Urine is a valuable biological sample. Urine protein level can be used as a universal indicator of kidney function. However, in terms of disease prediction, proteinuria mostly increases after a patient develops renal insufficiency and cannot be used for the early detection of IMN (39, 40). In studies exploring urine biomarkers, it was found that foot protein mRNA in urinary sediment can serve as a biomarker for podocyte detachment and functional impairment (41). Using the PPI network and verification with cellular samples from the second morning urine, we found that the genes *CALR*, *RACK1*, *CTNNB1*, *EEF1A1*, and *ATP5F1B* in PTCs all exhibited differences and disease correlations. Nevertheless, through verification using morning urine cellular RNA-seq, only *CALR* and *ATP5F1B* showed sustained differential expression. Hence, we believe that *CALR* and *ATP5F1B* have great potential as urine biomarkers for IMN.

Secondary morning urine was more accurate than morning urine (42). Through WGCNA analysis combined with RNA-seq of secondary morning urine cells, *CALR* was determined to be the factor most relevant to IMN and was positively correlated with Hb content. Additionally, as a key component of the peptide-loading complex, *CALR* ensures proper loading of cellular antigens onto MHC class I molecules and facilitates macrophage differentiation (43, 44). Current research indicates that mutations in exon 9 of CALR are promising targets for cancer immunotherapy (45). Therefore, we hypothesized that *CALR* is an important factor in the antigen presentation process of PTCs and potential immunotherapeutic targets for IMN.

Integrating existing clinical research enables a more comprehensive evaluation of the study’s significance (46). In an 11-year follow-up study, researchers observed that individuals with *CALR* mutations exhibited impaired renal function correlated with eGFR values, which aligns with our current findings (47). Additionally, *CALR* can influence hemoglobin (Hb) synthesis by activating the Janus kinase/signal transducer and activator of transcription (JAK/STAT) signaling pathway (48). Calcium homeostasis maintained by *CALR* is important in the erythropoietin (EPO) signaling process (49). Studies on patients with thrombocytosis have revealed a correlation between *CALR* mutations and EPO levels (48). The main reason for anemia in patients with IMN is that the kidneys gradually lose the ability to produce EPO during disease progression (50). The results indicated that *CALR* and Hb content showed a positive correlation. A decrease in *CALR* expression led to a reduction in the Hb content. Therefore, we hypothesized that the decrease in Hb content in the IMN is related to a reduction in EPO production caused by a decrease in *CALR* expression.

Ideal biomarkers should possess excellent predictive value, with a time course that can predict the time course of disease progression and a distinct cutoff value that enables the establishment of a remission state. Numerous studies have demonstrated that the integration of novel technologies significantly facilitates the identification of disease biomarkers (51). For instance, serum metabolomics has revealed distinct metabolic profiles and key biomarkers in IMN, with serum 25-hydroxyvitamin D being established as a predictive biomarker for clinical outcomes (52, 53). Research on gut microbial metabolites indicates that Lactobacillus species can ameliorate membranous nephropathy by modulating tryptophan-derived indole metabolites to suppress the aryl hydrocarbon receptor pathway (54). Furthermore, targeted modulation of Lactobacillus johnsonii has shown therapeutic potential (55). Urinary complement components demonstrate biomarker utility in IMN, potentially offering superior reflection of intrarenal inflammatory activity compared to systemic markers (56).

This study innovatively proposes urinary cellular RNA-seq technology to explore renal disease biomarkers at the transcriptome level. We not only identified sustained differential expression of *CALR* in urine but also validated its differential expression through immunohistochemistry, and qRT-PCR experiments. *CALR* can be used as a urinary biomarker for IMN and is closely correlated with the antigen presentation of PTCs and Hb content of patients with IMN. It should be noted that this study was limited to 17 IMN patients and 17 healthy volunteers; further validation through expanded sample sizes, inclusion of diverse renal disease types, and multicenter database analyses is required to substantiate our findings. Additionally, the molecular mechanisms underlying *CALR*’s role necessitate more comprehensive investigation through cytological experiments and animal models.

## 5 Conclusion

A comprehensive analysis was conducted by combining renal tissue scRNA-seq and morning and secondary morning urine cellular RNA-seq in IMN patients and healthy volunteers. This was combined with immunohistochemistry and qRT-PCR experiments for validation and *CALR*, a secreted factor closely related to IMN. *CALR* had excellent stability, which was verified in both morning and secondary morning urine samples. It plays a crucial role in the antigen-presenting function of PTCs and Hb production.

## Data Availability

The scRNA-seq raw data used in this study were deposited in the GEO database under accession number GSE241302, GSE171458, and GSE131685 and are publicly available for research purposes. The raw RNA-seq data of urine cells generated and analyzed during the current study are available from the corresponding author upon reasonable request.

## Footnotes

1
https://www.r-project.org/

## Supplementary material

Supplementary Figure 1 Single-cell data quality control chart.

Supplementary Figure 2 High-resolution source images of Figure 1A before panel assembly.

Supplementary Figure 3 High-resolution source images of Figure 1B before panel assembly.

Supplementary Figure 4 High-resolution source images of Figure 1C before panel assembly.

Supplementary Figure 5 High-resolution source images of Figure 3A before panel assembly.

Supplementary Figure 6 High-resolution source images of Figure 3B before panel assembly.

Supplementary Figure 7 High-resolution source images of Figure 3C before panel assembly.

Supplementary Figure 8 High-resolution source images of Figure 3D before panel assembly.

Supplementary Figure 9 Correlation chart of DEGs with GFR and Scr.

Supplementary Figure 10 Correlation of DEGs with Uric acid and blood fat.

Supplementary Figure 11 Immunohistochemical results of *CALR* in PLA2R^–^ MN (membranous nephropathy) renal tissues.

Supplementary Table 1 The primer sequences for β*-actin* and *CALR*.

Supplementary Table 2 Clinical information table for patients and healthy volunteers.
