# Supplementary figures and images for: Hemoglobin-associated CALR in proximal tubule cells can be used as a biomarker for idiopathic membranous nephropathy
Source: Front Med (Lausanne). 2025 Jun 11;12:1574852. doi: 10.3389/fmed.2025.1574852 (PMC12187741; doi:10.3389/fmed.2025.1574852)

A

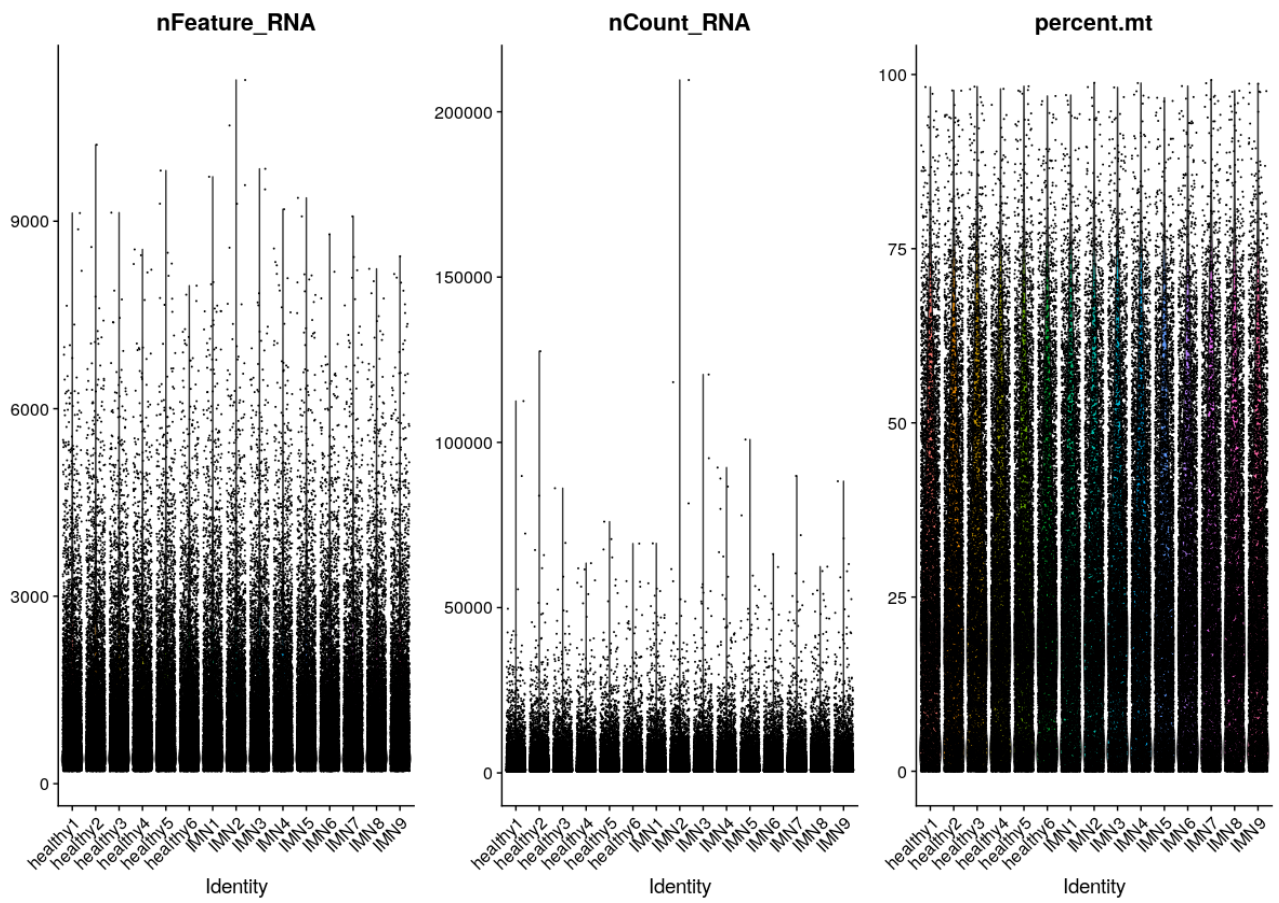

B

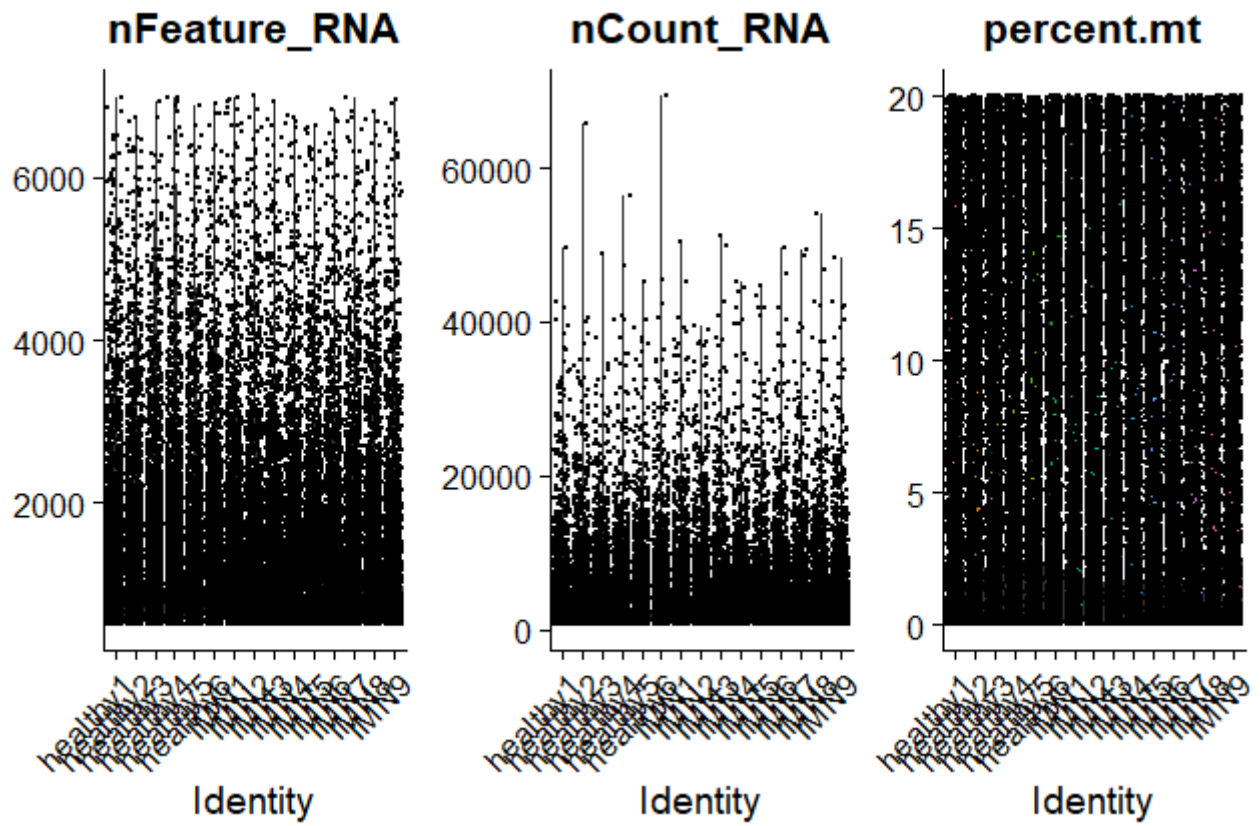

Supplement: Supplementary Figure 1 — Single-cell data quality control chart. [file Image_1.pdf]

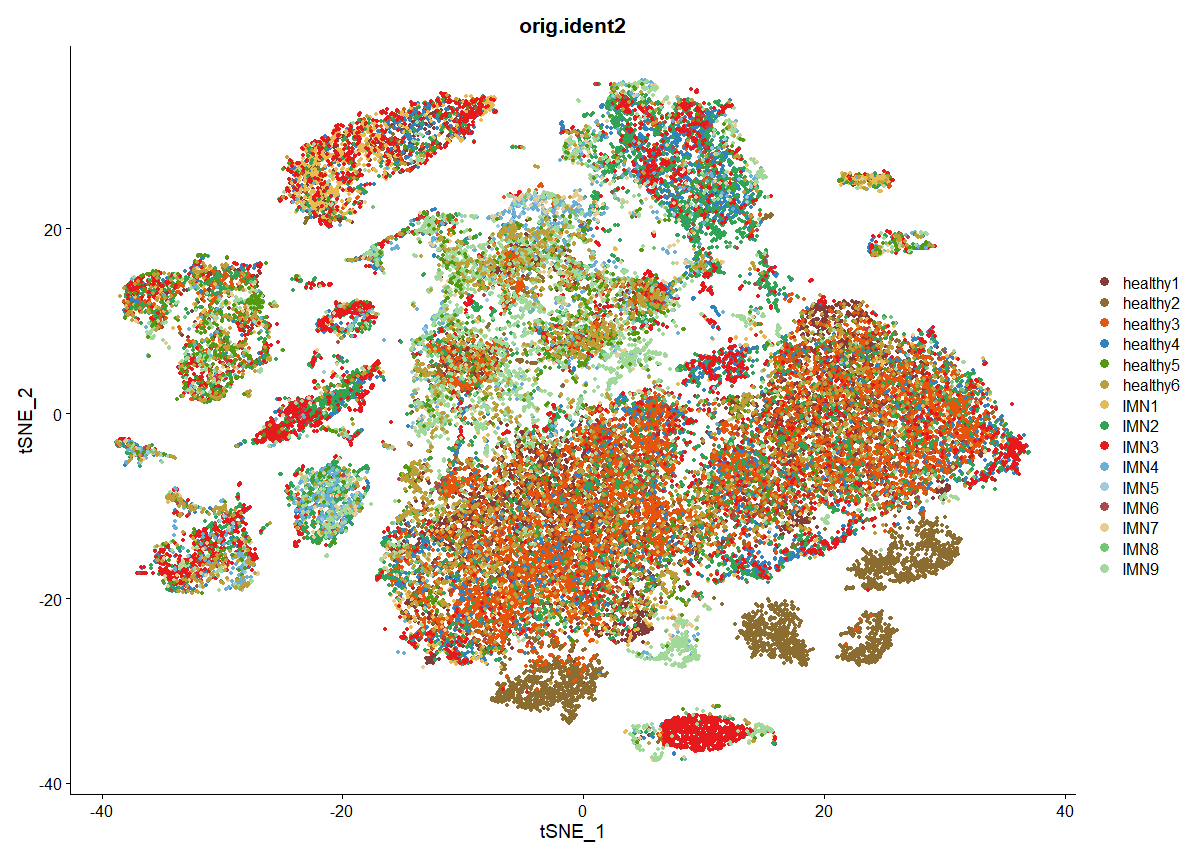

Supplement: Supplementary Figure 2 — High-resolution source images of Figure 1A before panel assembly. [file Image_2.tiff]

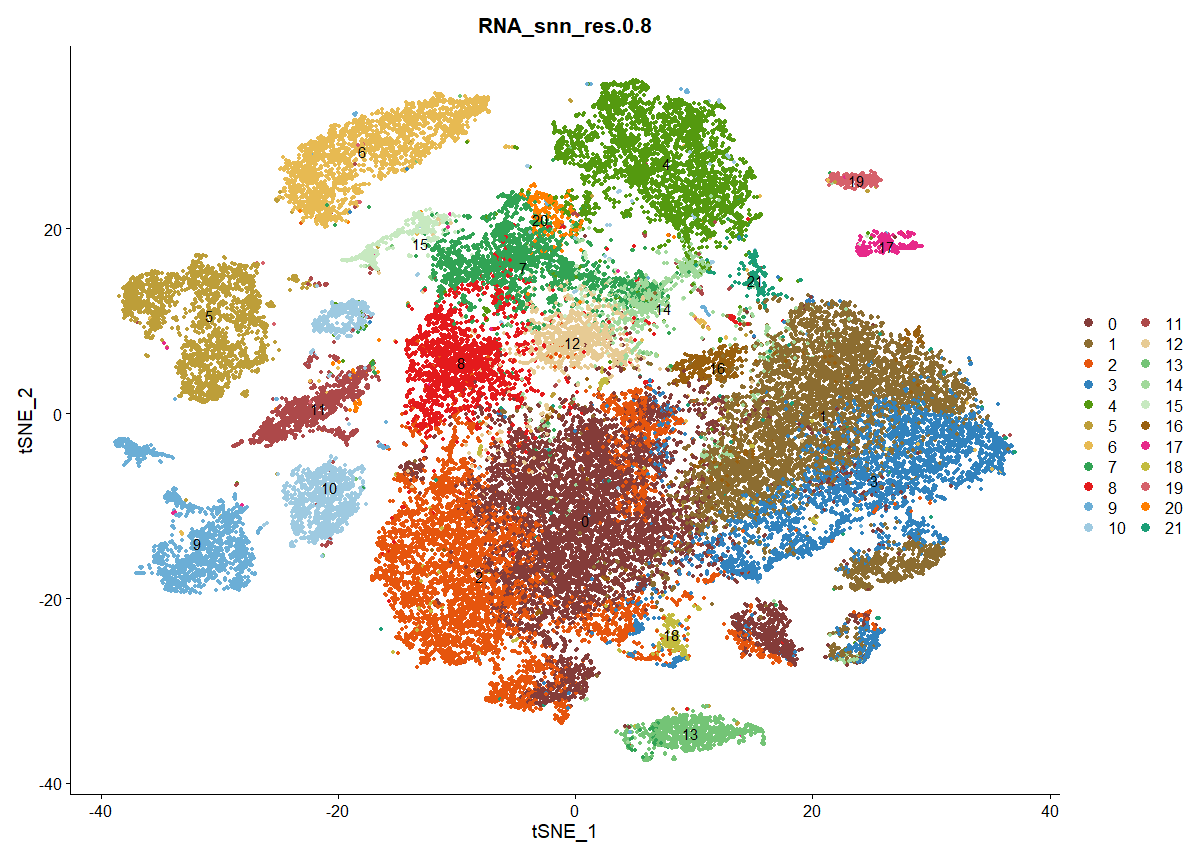

Supplement: Supplementary Figure 3 — High-resolution source images of Figure 1B before panel assembly. [file Image_3.tiff]

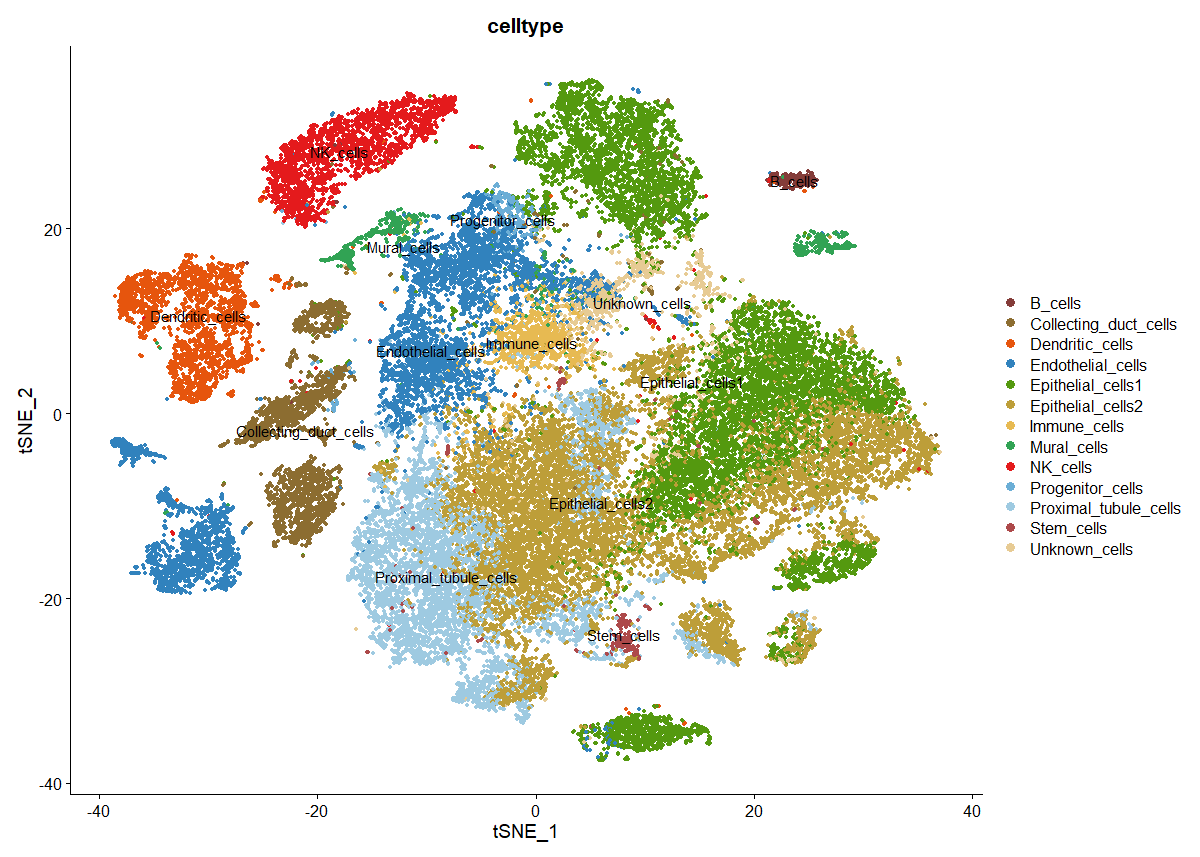

Supplement: Supplementary Figure 4 — High-resolution source images of Figure 1C before panel assembly. [file Image_4.tiff]

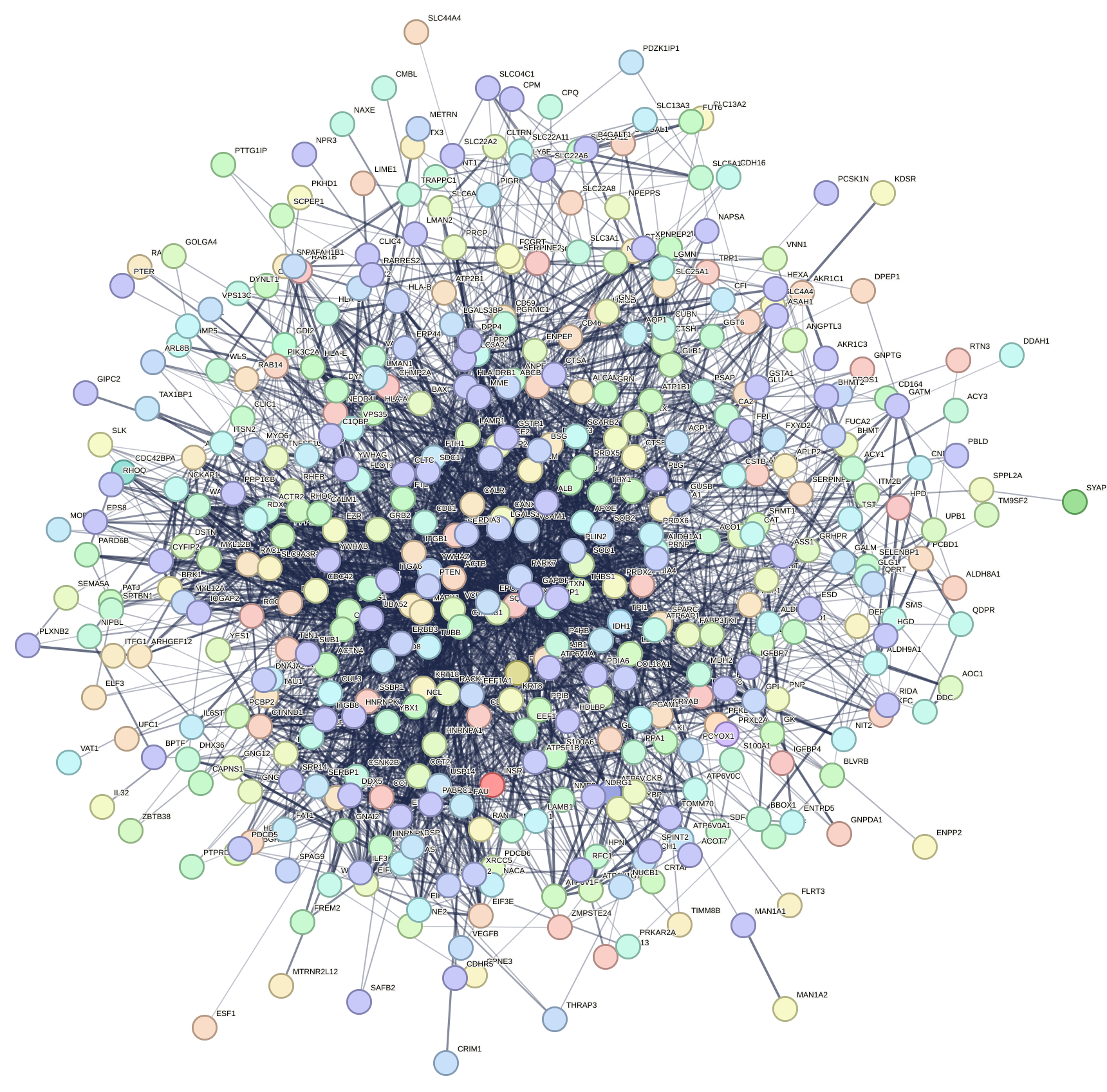

Supplement: Supplementary Figure 5 — High-resolution source images of Figure 3A before panel assembly. [file Image_5.png]

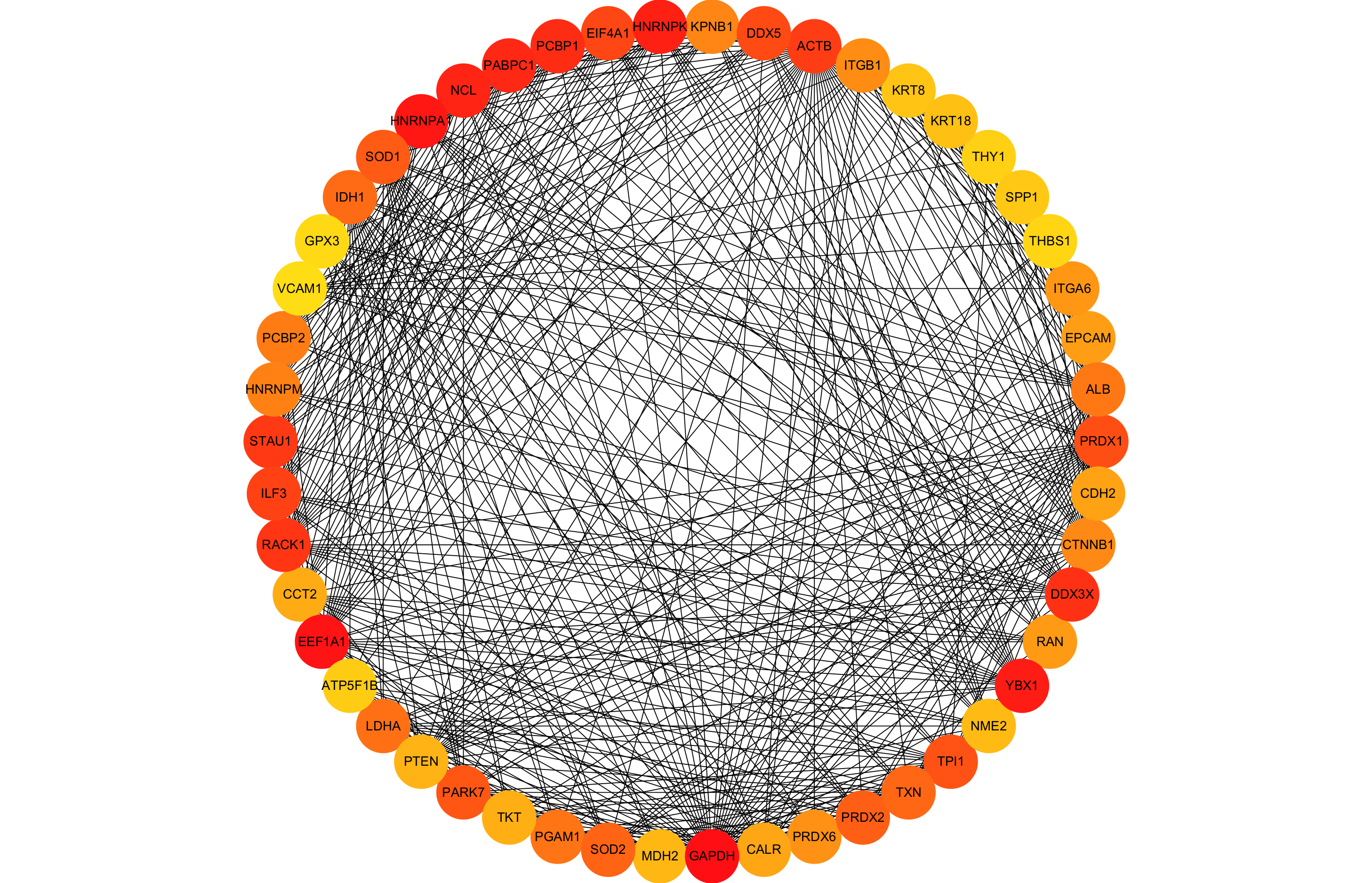

Supplement: Supplementary Figure 6 — High-resolution source images of Figure 3B before panel assembly. [file Image_6.png]

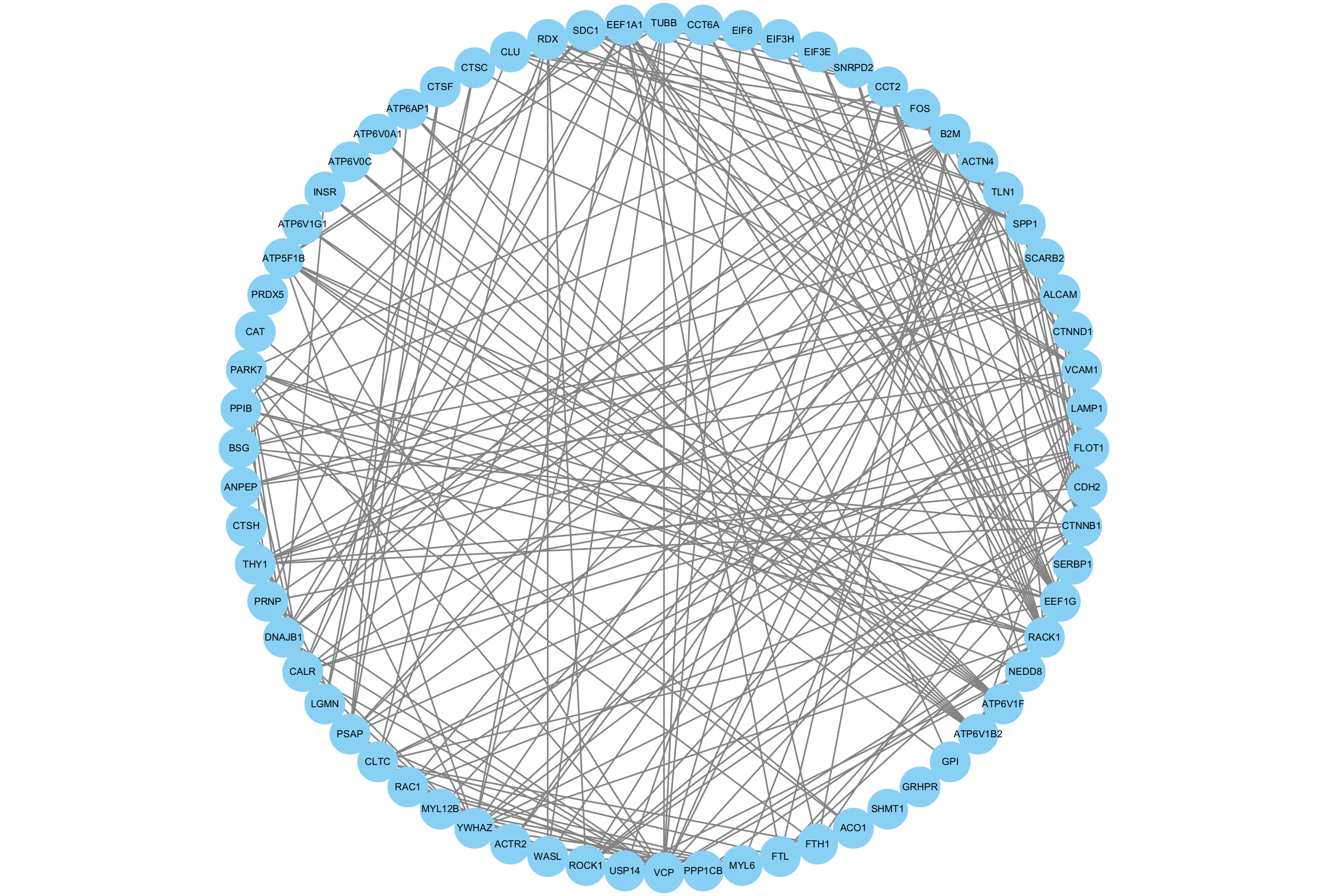

Supplement: Supplementary Figure 7 — High-resolution source images of Figure 3C before panel assembly. [file Image_7.png]

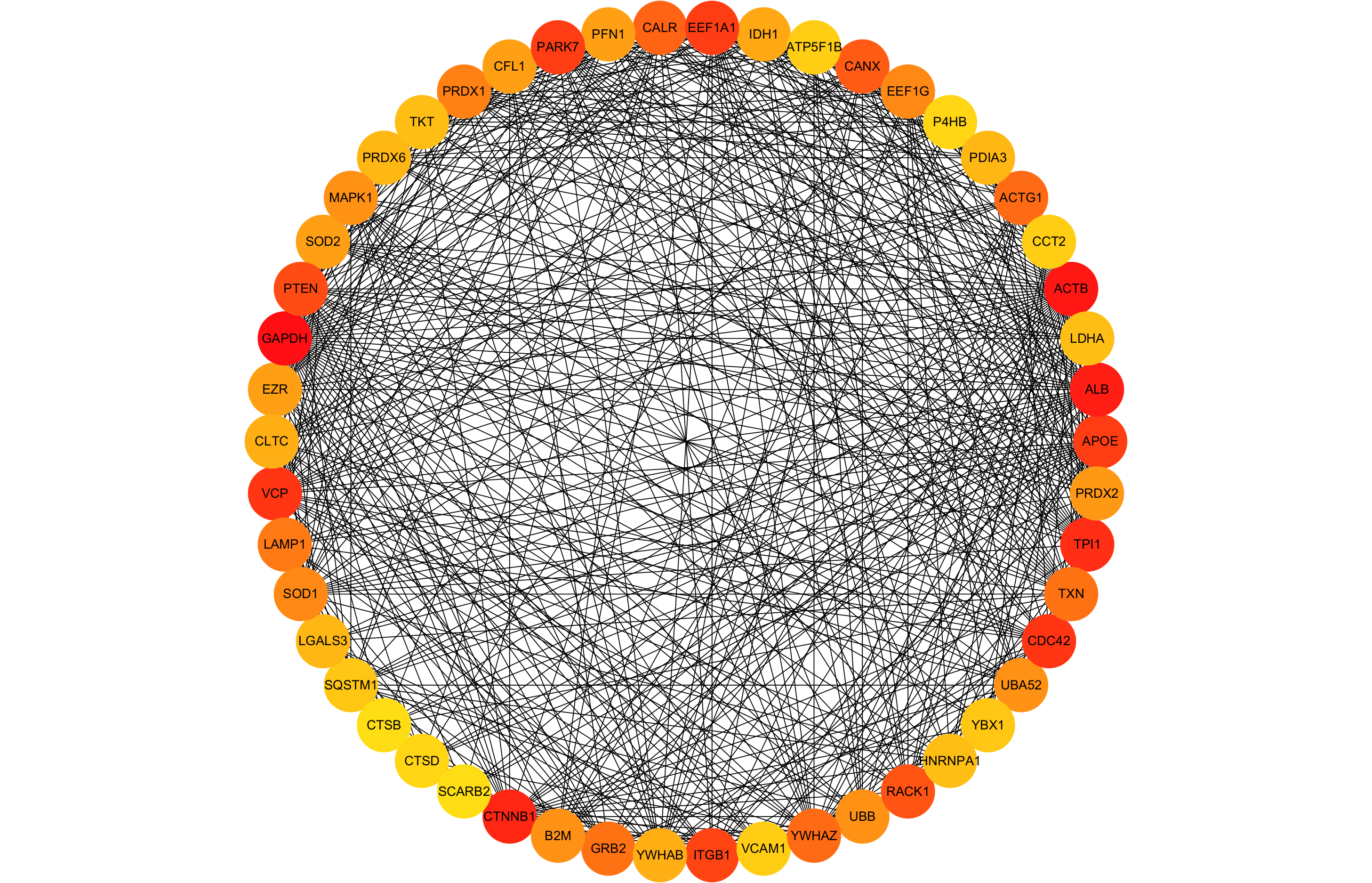

Supplement: Supplementary Figure 8 — High-resolution source images of Figure 3D before panel assembly. [file Image_8.png]

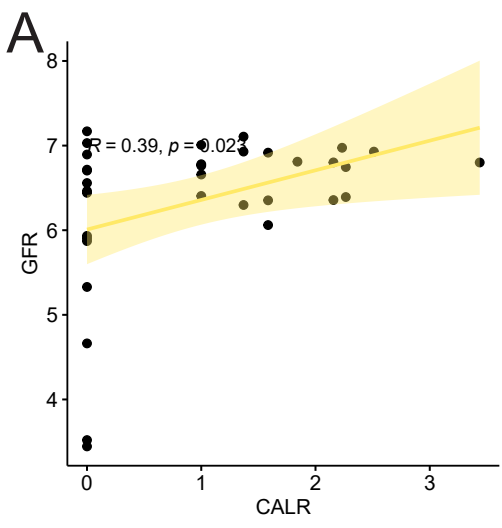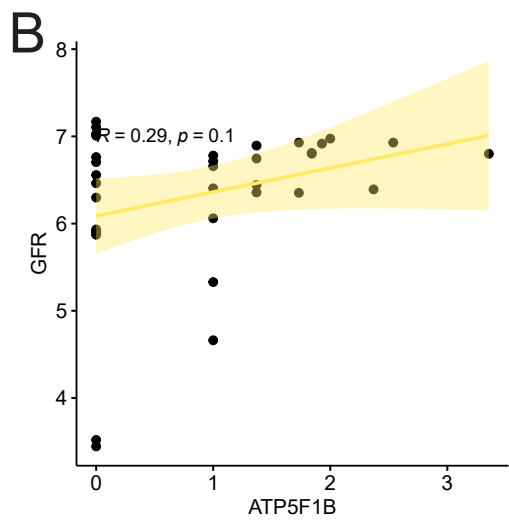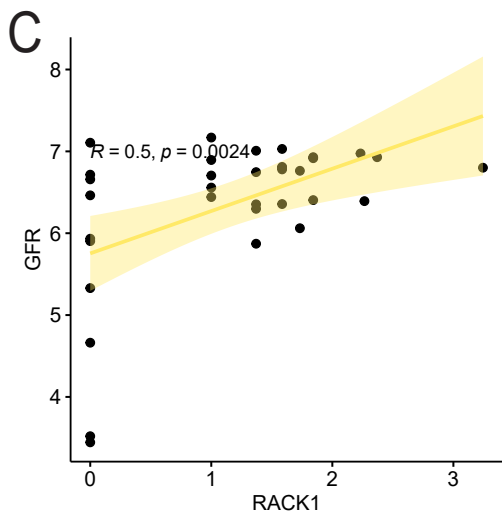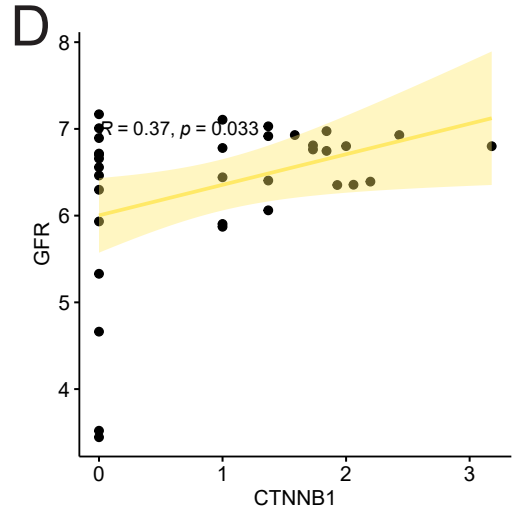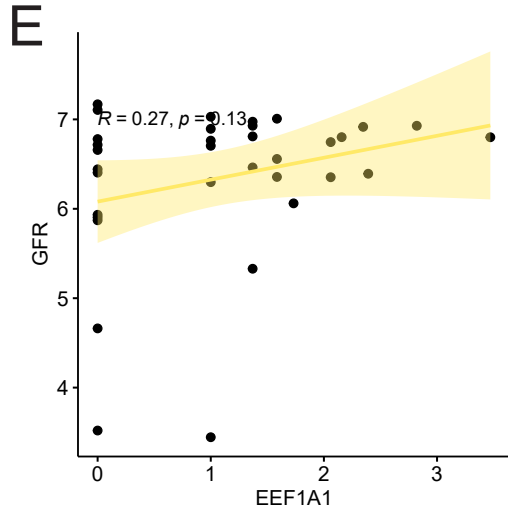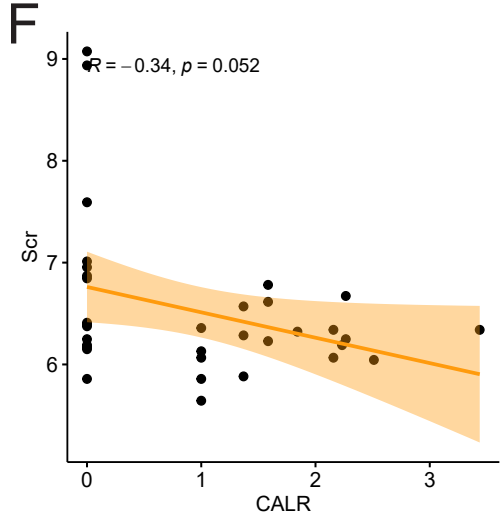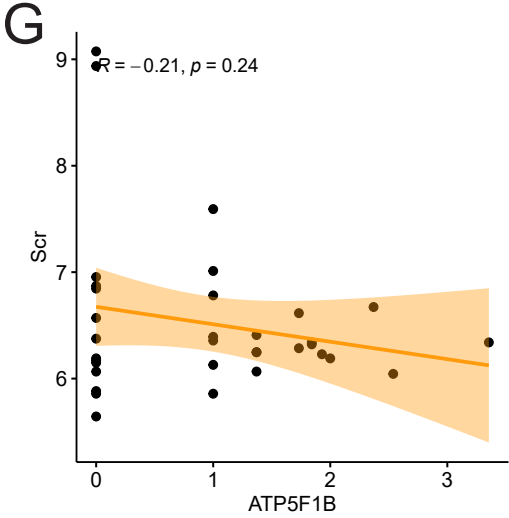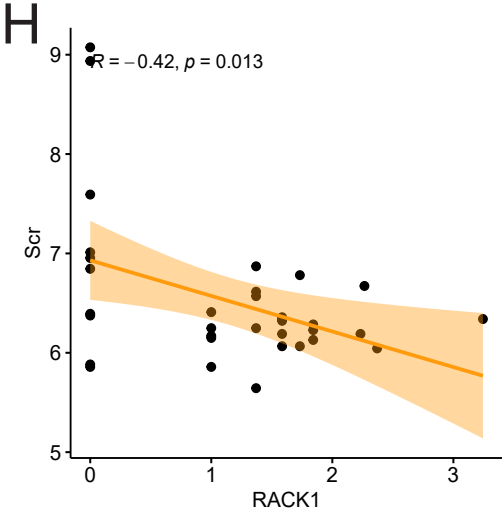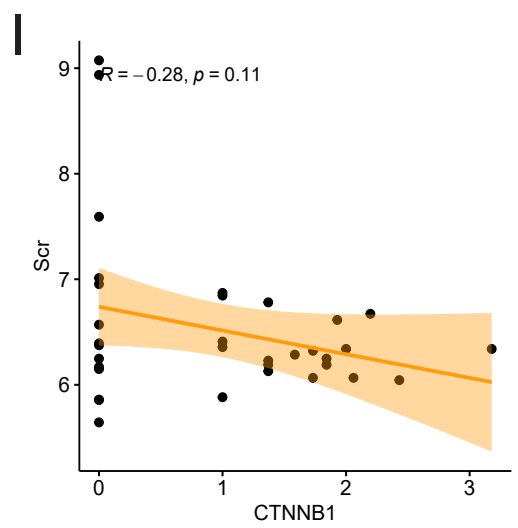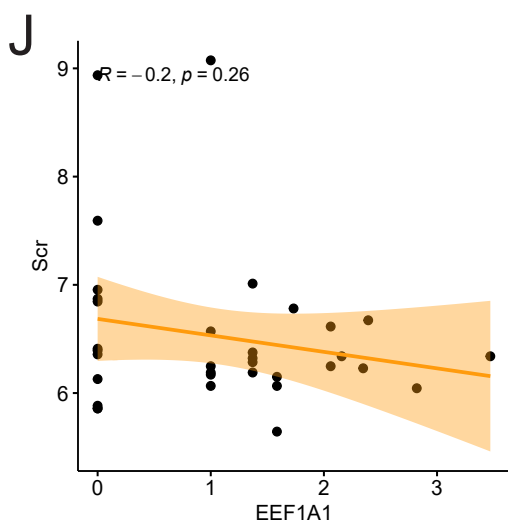

Supplement: Supplementary Figure 9 — Correlation chart of DEGs with GFR and Scr. [file Image_9.pdf]

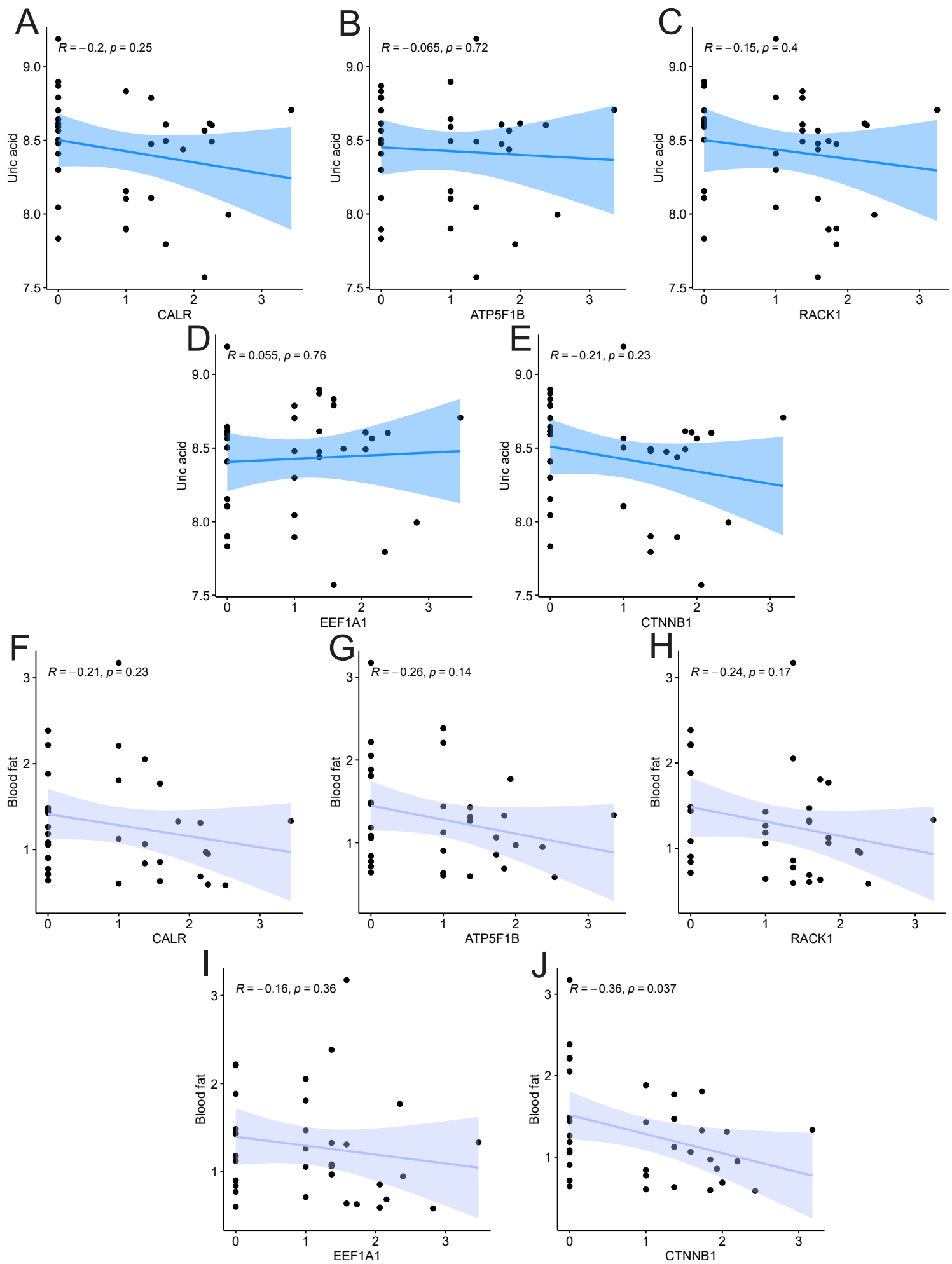

Supplement: Supplementary Figure 10 — Correlation of DEGs with Uric acid and blood fat. [file Image_10.pdf]

**10×**

**20×**

PLA2R-MN

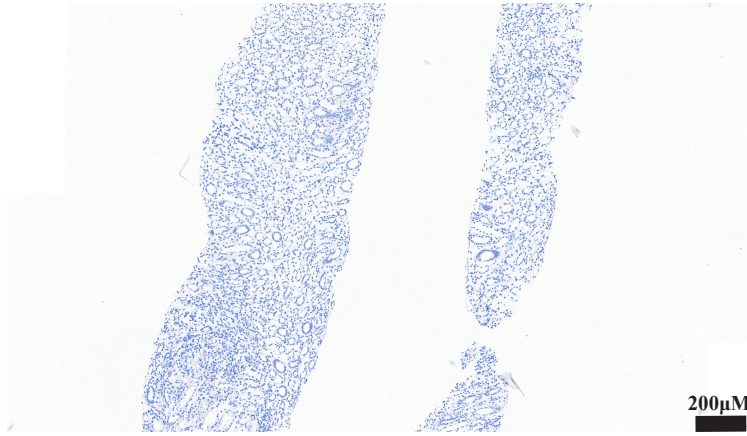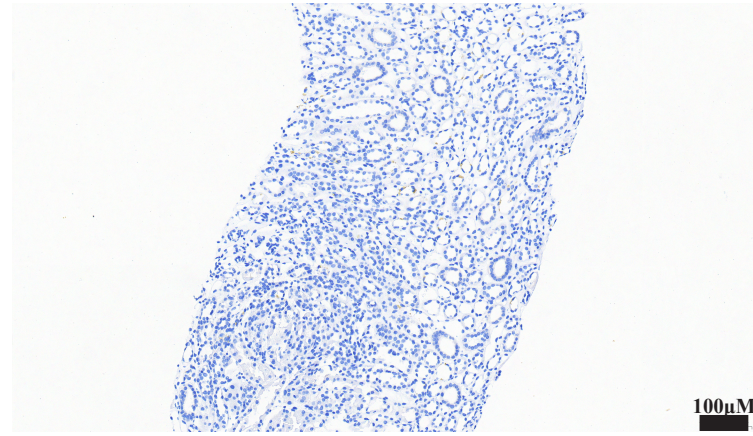

Supplement: Supplementary Figure 11 — Immunohistochemical results of CALR in PLA2R– MN (membranous nephropathy) renal tissues. [file Image_11.pdf]
