# Supplementary material for: Hemoglobin-associated CALR in proximal tubule cells can be used as a biomarker for idiopathic membranous nephropathy
Source: Front Med (Lausanne). 2025 Jun 11;12:1574852. doi: 10.3389/fmed.2025.1574852 (PMC12187741; doi:10.3389/fmed.2025.1574852)
Supplement: Supplementary Table 1 — The primer sequences for β-actin and CALR. [file Table_1.docx]

| patients/healthy volunteers | Renal tissue PAL2R | Hb | Scr | GFR | Blood fat | Uric acid |
| --- | --- | --- | --- | --- | --- | --- |
| IMN01 | + | 127 | 192 | 24.32 | 1.71 | 385 |
| IMN02 | + | 135 | 49 | 127.68 | 8.03 | 455 |
| IMN03 | + | 131 | 57 | 100 | 3.62 | 284 |
| IMN04 | + | 73 | 84 | 85.93 | 1.69 | 583 |
| IMN05 | + | 134 | 71 | 103.3 | 1.08 | 314 |
| IMN06 | + | 64 | 538 | 9.89 | 0.64 | 416 |
| IMN07 | + | 164 | 72 | 129.65 | 1.77 | 356 |
| IMN08 | + | 130 | 69 | 83.71 | 1.18 | 238 |
| IMN09 | + | 121 | 94 | 77.69 | 3.15 | 441 |
| IMN10 | + | 96 | 128 | 39.21 | 4.22 | 476 |
| IMN11 | + | 148 | 82 | 87.2 | 1.12 | 467 |
| IMN12 | + | 109 | 116 | 57.55 | 0.71 | 378 |
| IMN13 | + | 135 | 123 | 60.09 | 1.8 | 227 |
| IMN14 | + | 152 | 66 | 107.58 | 2.5 | 237 |
| IMN15 | + | 106 | 114 | 58.87 | 2.69 | 362 |
| IMN16 | + | 75 | 489 | 10.47 | 3.65 | 391 |
| IMN17 | + | 123 | 70 | 93.19 | 0.56 | 442 |
| healthy volunteer01 | NA | 145 | 75 | 106.35 | 0.51 | 359 |
| healthy volunteer02 | NA | 147 | 97 | 80.744 | 0.81 | 389 |
| healthy volunteer03 | NA | 188.8 | 65 | 120.83 | 0.5 | 254 |
| healthy volunteer04 | NA | 112 | 66 | 80.93 | 1.48 | 189 |
| healthy volunteer05 | NA | 164 | 80 | 110.53 | 0.61 | 378 |
| healthy volunteer06 | NA | 150 | 79 | 111.187 | 1.51 | 346 |
| healthy volunteer07 | NA | 176 | 74 | 119.812 | 2.41 | 221 |
| healthy volunteer08 | NA | 136.3 | 80 | 110.453 | 1.52 | 417 |
| healthy volunteer09 | NA | 145 | 101 | 82.986 | 0.93 | 388 |
| healthy volunteer10 | NA | 159 | 75 | 117.97 | 1.4 | 263 |
| healthy volunteer11 | NA | 153 | 81 | 108.881 | 0.52 | 274 |
| healthy volunteer12 | NA | 145.4 | 72 | 124.733 | 0.96 | 391 |
| healthy volunteer13 | NA | 136.8 | 109 | 65.758 | 0.55 | 360 |
| healthy volunteer14 | NA | 122.1 | 58 | 136.738 | 0.79 | 275 |
| healthy volunteer15 | NA | 120.1 | 57 | 142.891 | 1.27 | 339 |
| healthy volunteer16 | NA | 167 | 83 | 104.082 | 0.87 | 399 |
| healthy volunteer17 | NA | 149 | 77 | 120.851 | 1.09 | 355 |
