# Supplementary material for: Hemoglobin-associated CALR in proximal tubule cells can be used as a biomarker for idiopathic membranous nephropathy
Source: Front Med (Lausanne). 2025 Jun 11;12:1574852. doi: 10.3389/fmed.2025.1574852 (PMC12187741; doi:10.3389/fmed.2025.1574852)
Supplement: Supplementary Table 2 — Clinical information table for patients and healthy volunteers. [file Table_2.docx]

Supplementary Table 2. The primer sequences for *β-actin* and CALR

| Gene | Forward primer (5’→3’) | Reverse primer (5’→3’) |
| --- | --- | --- |
| β-actin | TCACCATGGATGATGATATCGC | CCACATAGGAATCCTTCTGACC |
| CALR | CCTGCCGTCTACTTCAAGGAG | GAACTTGCCGGAACTGAGAAC |
